# Thin-Layer-Agar-Based Direct Phenotypic Drug Susceptibility Testing on Sputum in Eswatini Rapidly Detects Mycobacterium tuberculosis Growth and Rifampicin Resistance Otherwise Missed by WHO-Endorsed Diagnostic Tests

**DOI:** 10.1128/AAC.02263-20

**Published:** 2021-05-18

**Authors:** E. Ardizzoni, E. Ariza, D. Mulengwa, Q. Mpala, R. de La Tour, G. Maphalala, F. Varaine, B. Kerschberger, P. Graulus, A. L. Page, S. Niemann, V. Dreyer, A. Van Deun, T. Decroo, L. Rigouts, B. C. de Jong

**Affiliations:** a Institute of Tropical Medicine, Antwerp, Belgium; b Infectious Diseases Service, Hospital Clinic-IDIBAPS, Barcelona, Spain; c Médecins Sans Frontières, Mbabane, Eswatini; d Médecins Sans Frontières, Geneva, Switzerland; e Ministry of Health (NRL), Mbabane, Eswatini; f Médecins Sans Frontières, Paris, France; g Epicentre, Paris, France; h Molecular and Experimental Mycobacteriology, Research Center Borstel, Borstel, Germany; i German Center for Infection Research (DZIF), Borstel, Germany; j Research Foundation Flanders, Brussels, Belgium; k University of Antwerp, Antwerp, Belgium

**Keywords:** resistance detection, TLA, Xpert MTB/RIF, tuberculosis, MDR, XDR, *rpoB* I491F mutant

## Abstract

Xpert MTB/RIF rapidly detects resistance to rifampicin (RR); however, this test misses I491F-RR conferring *rpoB* mutation, common in southern Africa. In addition, Xpert MTB/RIF does not distinguish between viable and dead Mycobacterium tuberculosis (MTB). We aimed to investigate the ability of thin-layer agar (TLA) direct drug-susceptibility testing (DST) to detect MTB and its drug-resistance profiles in field conditions in Eswatini. Consecutive samples were tested in parallel with Xpert MTB/RIF and TLA for rifampicin (1.0 μg/ml) and ofloxacin (2.0 μg/ml). TLA results were compared at the Reference Laboratory in Antwerp with indirect-DST on Löwenstein-Jensen or 7H11 solid media and additional phenotypic and genotypic testing to resolve discordance. TLA showed a positivity rate for MTB detection of 7.1% versus 10.0% for Xpert MTB/RIF. Of a total of 4,547 samples included in the study, 200 isolates were available for comparison to the composite reference. Within a median of 18.4 days, TLA detected RR with 93.0% sensitivity (95% confidence interval [CI], 77.4 to 98.0) and 99.4% specificity (95% CI, 96.7 to 99.9) versus 62.5% (95% CI, 42.7 to 78.8) and 99.3% (95% CI, 96.2 to 99.9) for Xpert MTB/RIF. Eight isolates, 28.6% of all RR-confirmed isolates, carried the I491F mutation, all detected by TLA. TLA also correctly identified 183 of the 184 ofloxacin-susceptible isolates (99.5% specificity; 95% CI, 97.0 to 99.9). In field conditions, TLA rapidly detects RR, and in this specific setting, it contributed to detection of additional RR patients over Xpert MTB/RIF, mainly but not exclusively due to I491F. TLA also accurately excluded fluoroquinolone resistance.

## INTRODUCTION

In 2019, about 10 million people globally developed tuberculosis (TB), and half a million people developed TB resistant to rifampicin (RR-TB) (1). Even if rifampicin (RMP) drug susceptibility testing (DST) coverage at TB diagnosis increased to 61%, a considerable number of patients with undetected RR-TB are still treated with an ineffective rifampicin-based treatment regimen. These patients are at high risk of treatment failure and continue spreading RR-TB (1).

To enhance RR detection, the End TB strategy recommends improving case detection and DST coverage also with the use of molecular techniques. The tests used most commonly to detect drug resistance are Xpert MTB/RIF (Cepheid, USA), which simultaneously detects Mycobacterium tuberculosis complex (MTB) and RR, and line probe assays (LPA) such as GenoType MTBDRplus, (Hain Lifescience, Germany) for isoniazid (INH) and RMP and MTBDRsl for fluoroquinolone (FQ) and second-line injectables (2). The rollout of Xpert MTB/RIF, also in peripheral laboratories, substantially decreased diagnostic delay (3, 4). However, these rapid molecular techniques miss specific *rpoB* mutants at positions outside the RR-determining region of the *rpoB* gene (RRDR), which are associated with equally poor treatment outcomes, as “common” *rpoB* mutants (5). One example is the I491F RR-conferring mutation among the so-called disputed mutations (6), which, in Eswatini, accounts for 56% of all RR-TB cases (7) and is reason for grave concern (8, 9). Furthermore, I491F is, in most of the cases, tested as false rifampicin sensitive (RS) in mycobacterial growth indicator tube (MGIT) phenotypic DST (pDST) and is only detected by sequencing of the entire *rpoB* gene or slow pDST on solid media, such as Löwenstein-Jensen (LJ) (9–11). Isolates with these mutations are also partially missed by other noncommercial methods, such as microscopic observation direct susceptibility testing (MODS), with a reported sensitivity of 75% (12).

TLA has previously been described as an affordable method to detect MTB (13, 14). This technique has limited costs (15) and can provide results considerably faster than indirect pDST methods. Direct thin-layer agar DST (referred to as TLA below) is not among the noncommercial methods recommended by WHO (16). However, when TLA is used for simultaneous MTB and drug resistance detection, clinical samples are inoculated immediately after decontamination on the medium with and without antibiotics (17,) avoiding the intermediate step of MTB isolation, which eliminates the need for high-level biohazard containment such as is needed for indirect pDST (18).

These characteristics make this test suitable for low-resource settings (19), although studies on its applicability in routine practice are still limited. In settings where the prevalence of the I491F mutation is low, TLA has shown similar overall performance to indirect MGIT-DST in terms of sensitivity, specificity, and turnaround time (15, 17). However, the ability of TLA to detect RR due to mutations outside the RRDR is unknown.

The primary objective of this study was to investigate the use of TLA as a direct pDST test for RR-TB detection in a peripheral laboratory, applied to smear-positive (Sm^+^) and smear-negative (Sm^−^) sputum samples in a setting with a high prevalence of the *rpoB* I149F mutation. In addition, we describe the test performance for detection of MTB and resistance to ofloxacin (OFX) as an indicator for FQ resistance, a key class of drug in the treatment of RR-TB. DST results were evaluated in comparison to a composite reference standard that included genotypic plus phenotypic testing.

## RESULTS

### MTB detection.

Between January 2014 and December 2016, 3,097 patients provided a total of 4,547 samples. The overall MTB positivity rate was 7.1% (322/4,547) for TLA and 10.0% (456/4,547) for Xpert MTB/RIF (Table 1). Among Sm^+^ samples, TLA positivity was 68.6% (138/201) versus 90.7% (176/194) for Xpert MTB/RIF, compared to 3.7% (153/4,107) versus 5.7% (234/4,130), respectively, for Sm^−^ samples (*P* < 0.0001 in both groups).

**TABLE 1:** Detection of Mycobacterium tuberculosis complex and nontuberculous mycobacteria for TLA versus Xpert MTB/RIF^*a*^

Xpert	TLA										Total
	Neg		Pos		NTM		Cont		Unknown		
	No.	%	No.	%	No.	%	No.	%	No.	%	
Total	3,857	84.8	322	7.1	11	0.2	255	5.6	102	2.2	4,547
Neg	3,536	91.2	32	0.8	10	0.3	218	5.6	83	2.1	3,879
Pos	161	35.3	274	60.1	1	0.2	18	3.9	2	0.4	456
High	24	15	124	77.5	1	0.6	11	6.9	0	0	160
Medium	39	35.1	70	63.1	0	0	2	1.8	0	0	111
Low	39	39.4	56	56.6	0	0	3	3	1	1	99
Very low	59	68.6	24	27.9	0	0	2	2.3	1	1.2	86
Inconclusive	76	81.7	2	2.2	0	0	12	12.9	3	3.2	93
Unknown	84	70.6	14	11.8	0	0	7	5.8	14	11.8	119

a Xpert, XpertMTB/RIF; TLA, thin-layer agar; Neg, negative result; Pos, positive result; NTM, nontuberculous mycobacteria; Cont, contamination; unknown, indeterminate result; inconclusive, an error or an invalid or no result; no., number of samples. High, medium, low, and very low indicate the level of positivity.

Among Xpert MTB/RIF-negative or inconclusive samples (*n* = 3,972), the relative gain of TLA for detection of MTB, when performed as a follow-on test after Xpert MTB/RIF, was 7.5% (34/456), while the reverse, the relative gain of Xpert MTB/RIF over TLA for MTB detection, was 55.9% (180/322).

The median turnaround time (TAT) between sample collection and TLA inoculation was 4 days (interquartile range [IQR], 2 to 7), while the median time from inoculation to TLA positivity was 11 days (IQR, 7 to 19).

When inoculation was performed within 4 days, or from 5 days or later from sample collection, the TLA positivity rate for Sm^+^ samples decreased from 72.6% to 59.0% (*P* = 0.053), while the negative rate increased from 20.1% to 28.3.8% (*P* = 0.16), though it did not reach statistical significance. Contamination varied from 7.4% to 12.1% (*P* = 0.27).

### Results available for evaluation of TLA for detection of drug resistance.

**FIG 1:**
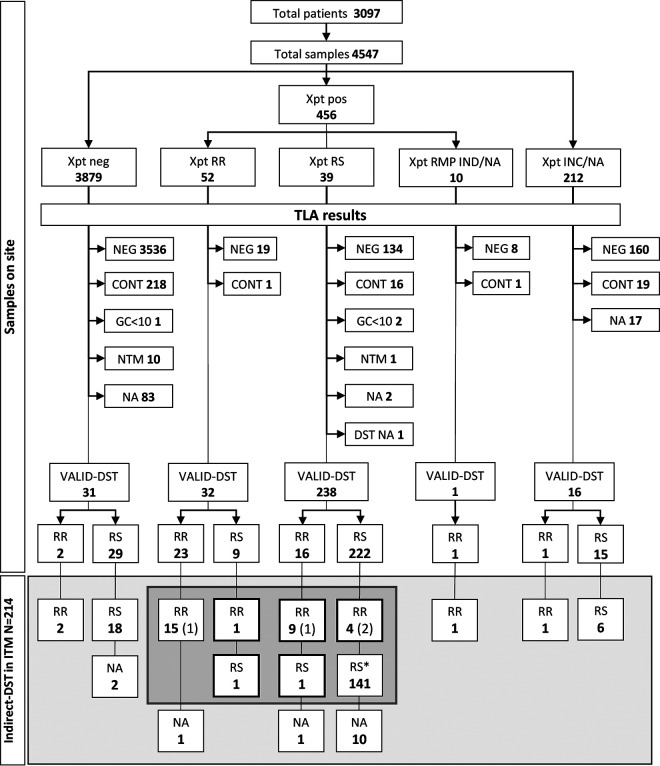
Phenotypic rifampicin resistance testing results from the site (direct, TLA) and the reference laboratory (indirect, LJ). Xpt, Xpert MTB/RIF; INC, result inconclusive (error, invalid, no result); RMP, rifampicin; GC, growth control; NTM, nontuberculous mycobacteria; IND, indeterminate; NA, not available; (*n*), administrative errors excluded; for details refer to Table S1. In the gray area, results for 172 isolates with indirect-DST, Xpert, and TLA results available, including administrative errors; *, 100 isolates selected for WGS, in addition to all RR detected by any method.

Out of 322 MTB-positive TLA plates, 214 (66.5%) had the corresponding LJ slants sent to the Institute of Tropical Medicine (ITM) (Fig. 1). At ITM, 200 (93.5%) of the isolates grew after subculturing. Four (2.0%) isolates were classified as administrative errors (Table S1 in the supplemental material), leaving 196 (98%) isolates available for analysis.

### RMP resistance detection.

Of 196 isolates included and with valid indirect-DST results at ITM, 168 (85.7%) had a valid initial Xpert MTB/RIF result. For another 28 (14.3%) samples, Xpert MTB/RIF results were either indeterminate or not available, while TLA was concordant with indirect-DST (Table 2).

**TABLE 2:** Rifampicin test results of TLA and Xpert MTB/RIF against composite reference standard

RMP result by CRS^*a*^	No. of isolates	*rpoB* target (Sanger sequencing/WGS)	Results of:				LJ-MIC (μg/ml)	MGIT DST	TLA final interpretation	TLA-Xpert concordance
			LJ	Xpert	TLA RMP DST	MTBDRplus				
RMP-R (*n* = 28)	1	M434I, I491F/M434I, I491F^*c*^	R	R	R	na	na	na	True RR	Concordant
3	na/H445L	R	R	R	na	na	na	True RR	Concordant
8	na/S450L	R	R	R	na	na	na	True RR	Concordant
	2	WT/WT^*d*^	R	R	R	na	na	na	True RR	Concordant
	1	H445D/H445D	R	Neg	R	na	na	na	True RR	na
		1	I491F, S450L, M434I/I491F, S450L, M434I^*c*^	R	Neg	R	na	na	na	True RR	na
1		I491F/I491F^*c*^	R	IND	R	na	na	na	True RR	na
	1	S450L/S450L	R	na	R	na	na	na	True RR	na
	5	I491F/I491F	R	S	R	WT	160 to >320^*b*^	3S, 2R	True RR	Discordant
	1	H445L, WT/WT^*f*^	R	S	R	WT	160	S	True RR	Discordant
		1	V170F/V170F	R	S	R	WT	160	R	True RR	Discordant
1		WT/WT^*e*^	R	S	R	WT	160	R	True RR	Discordant
1	S450L/S450L	R	R	S	delWT, MUT3	160	R	False RS	Discordant
1	WT/WT^*e*^	R	S	S	WT	160	R	False RS	Concordant
RMP-S (*n* = 168)	99	na/WT	S	S	S	na	na	na	True RS	Concordant
6	WT/nd	S	S	S	na	na	na	True RS	Concordant
36	na/na	S	S	S	na	na	na	True RS	Concordant
24	na/na	S	na	S	na	na	na	True RS	na
1	WT/WT	S	R	S	WT	20	S	True RS	Discordant
1	WT/WT	R	S	S	WT	20	S	True RS	Concordant
1	WT/WT	S	S	R	WT	20	S	False RR	Discordant

a CRS, composite reference standard; WGS, whole-genome sequencing; Xpert, Xpert MTB/RIF; LJ, Löwenstein-Jensen media; TLA, thin-layer agar; RMP, rifampicin; MGIT, mycobacterial growth indicator tube; na, not available; IND, RMP indeterminate; Neg, negative; nd, not determined.

b One result invalid.

c Isolates with I491F mutations either detected also by Xpert or without Xpert result.

d Paired isolates with *rpoB* sequencing WT results.

e P631S mutation in the *ponA1* region.

f rpoB H445L plus L452P detected at low-frequency mode.

After resolution versus the composite reference standard, a total of 168 (85.7%) isolates were finally classified as RS and 28 (14.3%) RR. Of the 168 samples with valid Xpert MTB/RIF results, 157 (93.5%), were concordant between Xpert MTB/RIF and TLA (142 RS and 15 RR), while 11 samples (6.5%) had discordant results between the two tests (Table 2). Of the 142 RS TLA and Xpert MTB/RIF concordant samples, one was RR by indirect-DST, making a total of 12 (6.1%) discordances for the 196 isolates tested between any two of the three tests.

With Xpert MTB/RIF, 9 (5.4%) isolates were falsely reported as RS, and one falsely showed RR, giving a sensitivity of 62.5% (15/24; 95% confidence interval [CI], 42.7 to 78.8) and specificity of 99.3% (143/144; 95% CI, 96.2 to 99.9). On TLA, RR was missed in two isolates, and in another isolate, TLA falsely showed RR. Hence, the sensitivity of TLA to detect RR was 93.0% (26/28; 95% CI, 77.4 to 98.0), and specificity was 99.4% (167/168; 95% CI, 96.7 to 99.9).

Most discrepancies between TLA and Xpert MTB/RIF RMP results were due to the presence of non-RRDR mutations outside the Xpert MTB/RIF target (five I491F and one V170F mutation) or wild-type (WT) *rpoB* genes with variable phenotypic results. All non-RRDR mutations were detected by TLA and had an RMP minimal inhibitory concentration (MIC) of ≥160 μg/ml. While V170F in one isolate was detected by MGIT, only 2 of 5 I491F mutations were detected by the liquid medium (Table 2).

In addition to the five I491F mutants found among TLA-Xpert MTB/RIF-discrepant results, another three I491F mutants were detected; one I491F in combination with M434I was RR by all tests, and two (one showing I491F mutation alone and one in combination with M434I and S450L) had no Xpert MTB/RIF RMP DST results (MTB not detected or RMP indeterminate). So, in total, 8/28 (28.6%) of the confirmed RR isolates had the I491F mutation, all of them showing also an S315T mutation in *katG* by whole-genome sequencing (WGS).

Two paired isolates from the same patient were RR by all phenotypic tests and Xpert MTB/RIF yet had a WT *rpoB* sequence (Table 2). Two additional isolates were consistently phenotypic RR (indirect-DST LJ, MIC LJ, MGIT, and TLA in one isolate), while all molecular assays (Xpert MTB/RIF, LPA, Sanger sequencing, and WGS) suggested a WT *rpoB* gene. In both isolates, WGS detected a P631S mutation in the *ponA1* region (Table 2). One isolate carrying the H445L-elusive mutation was RS by Xpert MTB/RIF and RR by indirect-DST LJ.

**FIG 2:**
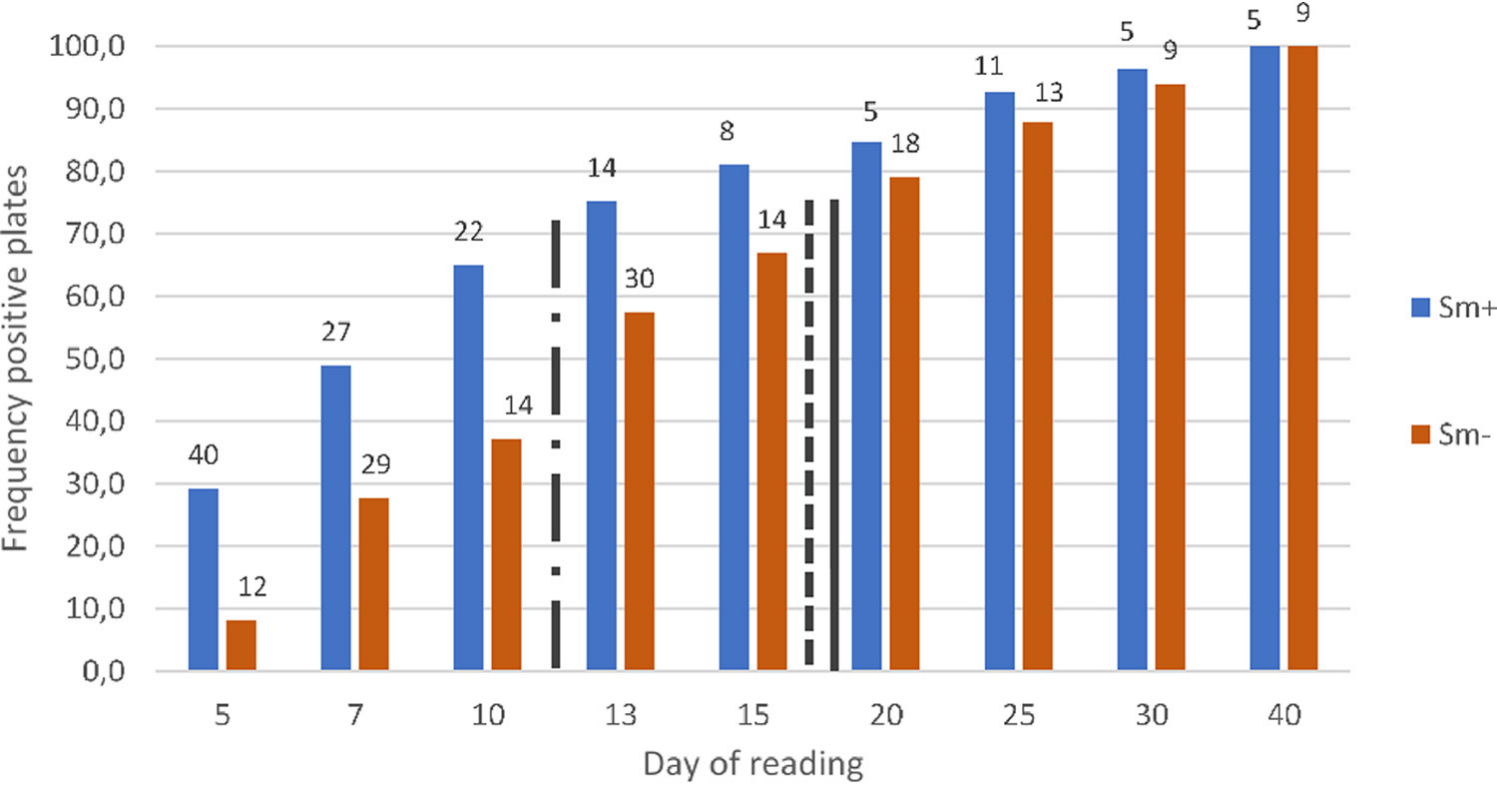
Number of positive plates and cumulative percentage per day of reading. Sm^+^, sputum smear microscopy positive; Sm^−^, sputum smear microscopy negative. Indication of median positivity and drug susceptibility results for rifampicin-susceptible (S) isolates (black line with dashes and dots), rifampicin-resistant isolates with mutations different from I491F (solid black line), and rifampicin-resistant isolates with I491F mutation only (dashed black line).

Overall, the median time to detect RR was 18.4 days, similar to 17.0 days for resistance conferred by I491F alone (*P* = 0.8) and 18.3 days for isolates carrying other RR-conferring mutations, but significantly longer than the 12.2 days for RS isolates (*P* = 0.03) (Fig. 2).

### OFX drug resistance detection.

Overall, of the 196 isolates included in the analysis, 185 had valid indirect-DST results in ITM. One isolate carrying mutation D94N was OFX-R (2.0 μg/ml) on indirect-DST and correctly detected by TLA. A total of 184 isolates were classified as OFX-S, all except one correctly identified by TLA (183/184; specificity 99.5%; 95% CI, 97.0 to 99.9) (Table 3), including three isolates that were OFX-R by indirect-DST but susceptible by molecular tests. One isolate was false OFX-R but WT for all molecular tests.

**TABLE 3:** Thin-layer agar results versus indirect-DST and composite reference standard for resistance to ofloxacin

OFX result by CRS^*a*^	No. of isolates	*gyrA/B* target (Sanger sequencing/WGS)	LPA target	Result of:		TLA final interpretation
				LJ (2.0 μg/ml OFX)	TLA (2.0 μg/ml OFX)	
OFX-R (*n* = 1)	1	na/D94N	na	R	R	True OFX-R
OFX-S (*n* = 184)	117	na/WT	na	S	S	True OFX-S
1	na/WT	na	S	R	False OFX-R
62	na/na	na	S	S	True OFX-S
1	WT/na	WT	S	S	True OFX-S
3	WT/WT^*b*^	WT	R	S	True OFX-S

a CRS, composite reference standard; TLA, thin-layer agar; OFX, ofloxacin; WGS, whole-genome sequencing; na, not available.

b For 1 isolate, WGS was not done.

## DISCUSSION

This study evaluated the TLA performance for MTB detection and direct DST for RMP, alongside OFX testing. In Nhlangano, a peripheral and low-resource setting, TLA showed a relatively high positivity rate for MTB detection, albeit below the ≥83% between smear-positive samples reported by other studies (13, 14). In our study culture, positivity was slightly affected by a delay in sample processing, while the contamination rate did not significantly increase. These results suggest that TLA may be suitable in laboratories at peripheral levels, where samples collected from remote areas are transported for testing. TLA performed excellently to detect RR after a median of 18.2 days.

In our study, more than 80% of the discordances between initial Xpert MTB/RIF and TLA were resolved in favor of TLA, which also correctly detected all I491F mutations, which accounted for almost half of all discordances.

In Eswatini, the prevalence of I491F is reason for concern. This mutation is regularly missed by Xpert MTB/RIF, a limitation that persists in the new version, Xpert MTB/RIF Ultra (20), with patients misdiagnosed as having RS-TB and receiving repeated rounds of ineffective first-line treatment. The national drug resistance survey carried out in Eswatini from 2017 to 2018 (7) has shown that the prevalence of I491F in MDR isolates has reached 56% compared to 30% detected by the survey from 2009 (9, 11). In our study, I491F caused 28.6% of all RR, although our findings may not be representative of the entire country. Indeed, WGS analysis showed that isolates with I491F mutations belong to a particular outbreak clone that evolved over time and acquired further resistance to first- and second-line drugs (21, 22). Thus, isolates with this particular mutation, not detected by standard diagnostic tests, are an enormous public health problem. To improve rapid detection of these missed RR cases, the new algorithm proposed by the National TB Program in 2019 includes starting empirical multidrug-resistant TB (MDR-TB) treatment for all detected INH-R cases while pursuing pDST on solid medium and sequencing of the *rpoB* gene to determine RR (23). This approach is supported by our results, where all the isolates with I491F mutations also carried mutations in the *katG* gene, correlated with INH resistance. In this algorithm, TLA could play a role to rapidly detect these RR cases at a peripheral laboratory equipped for moderate hazardous containment (biosafety level 2 [BSL2]) (18) while waiting for sequencing results.

Partial fitness loss for isolates carrying the I491 mutation has been proposed as a reason for false RS results in MGIT due to the short incubation time (maximum, 14 days) (5, 10). Despite the relatively short turnaround time on TLA (median, 17.0 days), none of the I491F strains were false RS on TLA compared to three of the five MGIT tested. The time to detection for the I491F mutants did not differ from the ones carrying other RR-conferring mutations, albeit collectively, the *rpoB* mutants grew significantly slower than *rpoB* wild-type isolates on primary isolation.

Besides I491F, another non-RRDR mutation (V170F) detected in one isolate was missed by Xpert MTB/RIF and LPA, yet was detected by TLA. This mutation is globally less frequent (24), is reliably detected by pDST, including MGIT, and has not yet led to known microepidemics.

In our study, one (16.7%) of the isolates carrying an H445L-elusive mutation, also showed a WT minority population. RR was detected by TLA, LJ-based pDST, and Sanger sequencing, but missed by rapid molecular tests and MGIT. The mutation was detected by WGS only at low frequency. Indeed, heteroresistance may be the cause of false-susceptible results. Tests have different limits of detection for minority populations, as low as 1% for phenotypic tests, 5% for MTBDRplus, 20 to 40% for Xpert MTB/RIF classic, 20% for Sanger sequencing (25), and 1% for WGS (26), depending on coverage depth. In addition, detection of minority populations and variants causing resistance can be challenged by preselection during primary culture isolation and multiple subcultures (27).

Surprisingly, two isolates, phenotypically RR by indirect-DST, MGIT, LJ-MIC, and, in one case, by TLA, were WT *rpoB* by Sanger sequencing, while WGS showed presence of the mutation P631S in *ponA1*. Polymorphisms in this region, which encode a protein involved in mycobacterial growth and cell wall synthesis, seem to constitute and be of advantage for growth in the presence of rifampicin and modify susceptibility to this drug (28, 29). Even if rare, the role of this mutation and other non-*rpoB* resistance-conferring mechanisms should be further investigated.

Of the three TLA OFX-S isolates found OFX-R on indirect-DST yet were WT by sequencing, two (paired isolates from the same patient) were borderline resistant by the indirect-DST, as they showed the same number of colonies in the drug-free and drug-containing tubes, one of them positive only with three colonies. Although these borderline results could be the cause of discordances, indirect-DST could not be repeated. For the third, no obvious explanation for the discrepancy could be found.

Laboratories in peripheral settings usually lack high-risk TB facilities with stable power supply, negative pressure, and complex equipment that is regularly maintained, which limits the implementation of phenotypic testing, especially indirect-DST (30). TLA direct DST, similarly to MODS, poses less biosafety risk, lowering requirements for moderate-risk facilities to contain biohazard potentially created by sample centrifugation and vortexing (18). In a head-to-head comparison, TLA showed to be superior to MODS in detecting resistance due to I491F mutation (12).

Our study is the first to test TLA for RMP and OFX in a high endemic setting at district level, although numbers did not suffice for full assessment of OFX performance.

A drawback pointed out for direct TLA DST is the lack of standardization of the inoculum (31), although we did not find an association of bacterial load (as determined by smear microscopy) and false DST results. Second, the direct nature of TLA testing complicates appropriate quality controls, for which fresh sputum is needed, as manipulation of strains would increase biosafety requirements. However, the use of samples spiked with avirulent strains, compatible with BSL2 hazard containment, could be considered for this purpose (32).

For 4 isolates (2.0%), discordant results could not be explained and, for this reason, were considered administrative errors. These errors are not infrequent in diagnostic laboratories (33) that handle large numbers of tests.

The TLA technique requires multiple readings of the plates, which is time-consuming. Our cumulative data suggest that the workload could be reduced by decreasing the number of readings, as readings at days 28 and 35 provided relatively low incremental yield. For future testing, reading at days 7, 14, 21, and 42 may be logistically more feasible, as well as the use of a redox indicator allowing for macroscopic reading, as suggested by recent studies (34, 35).

Our study presented some limitations. It was not possible to link on-site multiple samples collected from the same patient, so a per-patient analysis of the results was not possible. In addition, confirmatory tests were performed only on isolates showing discordances. TLA showed to correctly exclude resistance in all susceptible cases; however, our samples did not include a sufficient number of isolates resistant to OFX to fully evaluate TLA performance. In addition, molecular tests for concordant OFX tests were only partially available. In our study, OFX was considered an indicator of resistance to the class of FQs. The full performance of TLA for detection of FQ resistance, possibly including other FQs than OFX, should be assessed in settings with higher rates of FQ-R, or when TLA is used after Xpert RR diagnosis. Nevertheless, our study shows that TLA provides numerous advantages.

In settings with a high prevalence of I491 mutations, TLA could be very useful for patients with presumed RR-TB yet are Xpert MTB/RIF RS-TB. Especially in patients with INH-R and missed RR-TB, the use of an FQ-strengthened regimen, as recommended by WHO guidelines (36), would result in ethambutol and pyrazinamide as sole effective drugs, increasing the risk of developing extensively drug-resistant TB (XDR-TB) (37).

The relative gain for MTB detection by TLA when used after Xpert was limited, confirming that Xpert should be the first test for diagnosis (15, 17). However, TLA could be used for monitoring patients during treatment and also to detect amplification of RR-TB in patients failing first-line treatment, especially if missed at baseline. Validation of TLA for new and repurposed drugs would be the logical next step, given the WHO recommended pDST, as TLA could replace, in the flowchart, indirect-DST in patients found to have molecular evidence of RMP and/or INH resistance. The level of phenotypic resistance to bedaquiline that is conferred by *Rv0678* mutations is, for instance, largely unknown, and TLA could save weeks over indirect pDST and at a lower biosafety level.

In conclusion, TLA provides a relatively rapid diagnostic approach for detecting viable TB bacilli and simultaneous susceptibility testing for RMP, showing excellent sensitivity for the detection of RR due to the I491F mutation outside the RRDR that plagues Southern Africa. In the diagnostic algorithm of this setting, TLA could be used to test presumed RR patients yielding an RS result by Xpert MTB/RIF.

## MATERIALS AND METHODS

### Study patients and test flow.

**FIG 3:**
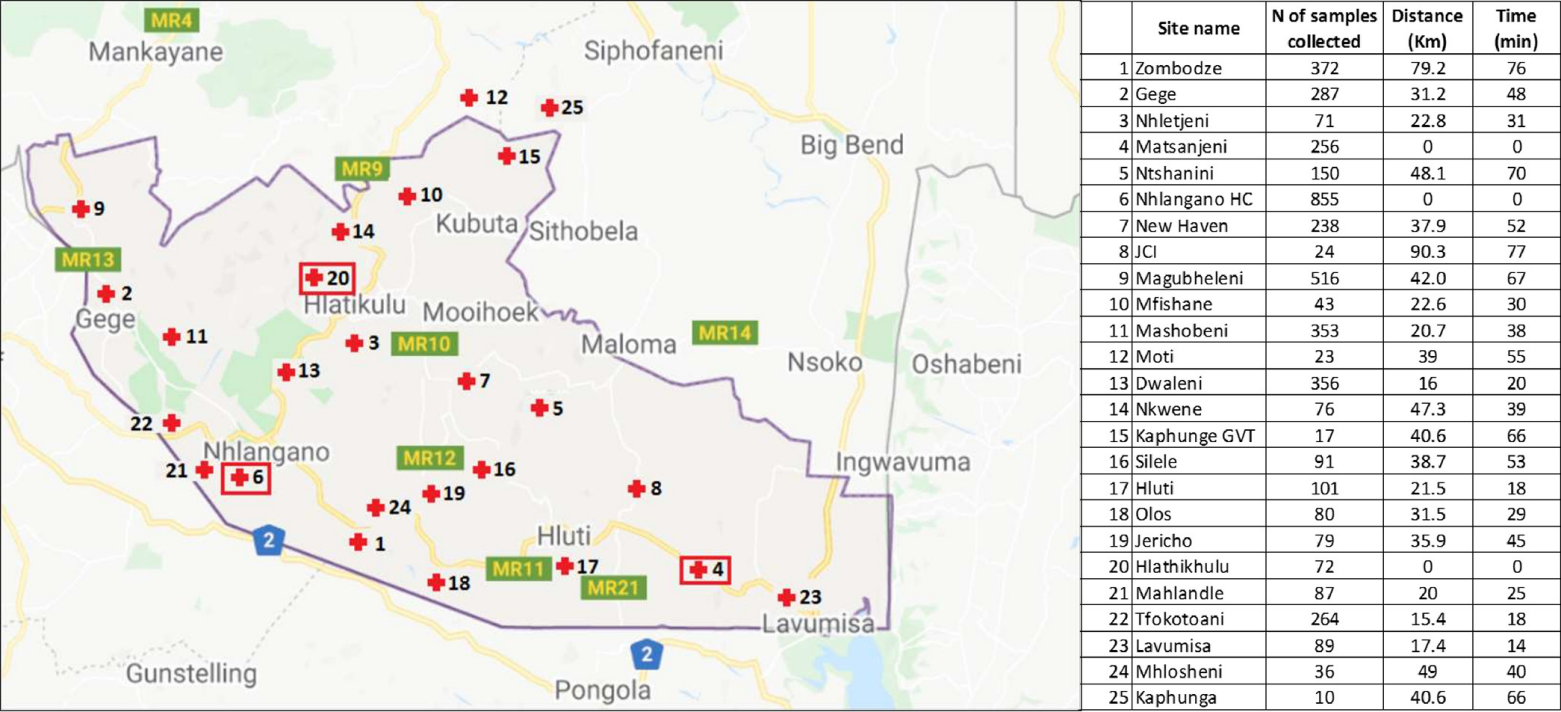
Sample collection sites in the Shiselweni region (Google map, modified). Distance and time of driving from the site of collection to the closest of the three TB laboratories (shown by red squares).

The study was conducted in the Nhlangano TB laboratory (NTBL), Eswatini, by Médecins Sans Frontières Switzerland and the Institute of Tropical Medicine (ITM), Antwerp, Belgium. The catchment area included 22 health clinics and 3 health centers (including Nhlangano) at up to 90 km of distance (map in Fig. 3), from where samples were sent to the NTBL in cold chain (2 to 8°C) without preservatives.

We included all samples collected from consecutive patients older than 15 years who presented signs of presumptive TB, did not start TB treatment in the previous month, and consented to participate in this study. As per routine practice, patients were asked to produce two good-quality sputum specimens (labeled as sample A and sample B) in 50-ml sterile screw-cap containers and, when collected at the clinics, sent at the health centers, where sample A was tested by Xpert MTB/RIF following Cepheid procedures. Any patients who were Xpert MTB/RIF positive for TB on sample A, regardless of the RMP result, had sample B sent to the National TB Reference Laboratory (NTBRL) in Mbabane for routine testing with liquid culture and pDST. In this case, sample C was collected for the purpose of this study. All leftovers from sample A, sample B, and sample C, if collected, were decontaminated and tested for the study with TLA, fluorescence microscopy (FM), Xpert MTB/RIF, and LJ culture at the NTBL. For any positive TLA plate inoculated with either sample A, B, or C, the corresponding LJ isolate was sent to ITM for extended phenotypic and genotypic testing. In case the LJ culture was contaminated or remained negative, an LJ subculture from the growth of the corresponding TLA plate was shipped.

### Laboratory methods at the NTBL.

We performed TLA using 4-quadrant polystyrene plates prepared at the NTBL. The medium contained 7H11 agar supplemented with a broad-spectrum antibiotic mixture to suppress contamination, as previously described (15, 17). TLA plates included drug-free growth control (GC), *p*-nitrobenzoic acid (PNB) (500 μg/ml), RMP (1.0 μg/ml), and OFX (2.0 μg/ml). The processed sputum sediment was resuspended with 2 ml phosphate-buffered saline (PBS), and two drops were inoculated per well.

During incubation at 5% CO_2_, plates were read at day 5, 7 10, 13, 15, 20, 25, 30, 35, and 40 as previously described (15), with few modifications. In addition, the drug-containing wells were read on the day of GC positivity and on the next scheduled reading.

Plates were reported as positive for nontuberculous mycobacteria (NTM) in case of mycobacterial growth in the quadrant containing PNB, while no or poor growth (≤3 colonies) on PNB with a positive GC compartment was considered positive for MTB (38). Any growth on PNB was tested with the MPT64 Ag test (SD Bioline).

### Laboratory methods at the reference laboratory.

Upon receipt of isolates at ITM, fresh subcultures were made on LJ medium, and indirect-DST was performed using the proportion method on LJ for RMP (40 μg/ml) and on 7H11 for OFX (2.0 μg/ml). Indirect solid DSTs were read blindly with respect to TLA.

All isolates with a discordant RMP result between any two of three tests (Xpert MTB/RIF, TLA, or indirect-DST) were further tested by MTBDRplus and had pDST done in MGIT (RMP 1.0 μg/ml), and the MIC for RMP was determined on LJ (10 to 160 μl/ml). All isolates showing RR on any test and 100 isolates showing RS on all tests were investigated by Sanger sequencing of the *rpoB* target at ITM and/or by whole-genome sequencing (WGS) performed at the Borstel Research Center (Germany) as previously described (39, 40). To constitute a composite reference standard for RMP resistance, MIC prevailed on pDST, and resistance found on any of the genotypic tests overrode any phenotypic result.

In the case of discordance between TLA and indirect-DST for OFX, isolates were investigated by LPA MTBDRsl and sequencing (target genes and/or WGS). As composite reference standard, any resistance related to a high-confidence mutation found on any of the genotypic DST overrode results showing susceptibility on another test.

### Statistical analysis.

For all tests conducted at the NTBL, we calculated the MTB positivity rate as the number of samples showing MTB over the total number of samples tested. The relative gain of TLA for detection of MTB over Xpert MTB/RIF was calculated as the number of samples that were TLA positive but Xpert MTB/RIF negative or inconclusive (including error, invalid, or no result) divided by the total number of samples positive on Xpert MTB/RIF. The relative gain of Xpert MTB/RIF versus TLA was calculated as well. We also calculated the median turnaround time (TAT) between sample collection and inoculation and the median time from inoculation to each test result and TAT for DST availability stratified by RMP resistance.

For all isolates received at ITM, we calculated the sensitivity and specificity (with 95% confidence intervals [CIs]) of TLA to detect resistance for RMP and specificity for OFX against the composite reference standards. Implausible discordant results (e.g., non-RRDR mutation on WGS but detected by Xpert [41]) were considered administrative errors and excluded from the analysis.

All statistical analyses were performed with Stata 12 software (Stata Corporation, College Station, TX).

### Ethics approval.

The study protocol was approved by the Institutional Review Board of ITM, the Ethics Committee of the University Hospital of Antwerp, Belgium, and the Ethics Committee of Eswatini.
