# Supplementary material for: Thin-Layer-Agar-Based Direct Phenotypic Drug Susceptibility Testing on Sputum in Eswatini Rapidly Detects Mycobacterium tuberculosis Growth and Rifampicin Resistance Otherwise Missed by WHO-Endorsed Diagnostic Tests
Source: Antimicrob Agents Chemother. 2021 May 18;65(6):e02263-20. doi: 10.1128/AAC.02263-20 (PMC8315964; doi:10.1128/AAC.02263-20)
Supplement: Supplementary file 1 [file aac.02263-20-s0001.pdf]

Table S1 Results for six isolates classified as administrative errors

WGS		Xpert MTB/RIF	MDRTB plus	TLA DST (µg/ml)		LJ-DST (µg/ml)		LJ-MIC RMP (µg/ml)	MGIT-DST RMP
rpoB	**gyrA/B**			**RMP 1.0**	**OFX 2.0**	**RMP 40**	**OFX 2.0**		
I491F	WT	R	nd	R	S	R	na	na	na
S450L	WT	S	delWT,MUT3	R	S	R	na	160	R
H445L	WT	S	WT	S	S	R	S	160	R
WT	WT	S	WT	S	S	R	R	20	S

GS=whole genome sequencing; Xpert=Xpert®MTB/RIF; LJ=Löwenstein-Jensen; TLA=thin layer agar; RMP=rifampicin; MIC=minimal inhibitory concentration; MGIT=mycobacterial growth indicator tube; na= not available; DST=drug susceptibility testing

In the first isolate a non-RRDR mutation, I491F, was identified through WGS and identified as RR by Xpert®MTB/RIF, which theoretically is not possible.

In the second isolate WGS showed the presence of S450L, a high confidence mutation, but it was missed by Xpert®MTB/RIF.

The third isolate carried the H445L mutation, unexpectedly missed by rapid molecular techniques.

The fourth isolate, WT by WGS for rpoB was RMP-R by LJ-DST. The isolate was also discordant for OFX 2.0µg/ml.
